# Light absorption engineering of a hybrid (Sn_3_S_7_^2−^)_*n*_ based semiconductor – from violet to red light absorption

**DOI:** 10.1038/srep45822

**Published:** 2017-04-04

**Authors:** Mathias Salomon Hvid, Paolo Lamagni, Nina Lock

**Affiliations:** 1 Interdisciplinary Nanoscience Center (iNANO), Aarhus University, Gustav Wieds Vej 14, DK-8000 Aarhus C, Denmark; 2 Carbon Dioxide Activation Center (CADIAC), Interdisciplinary Nanoscience Center (iNANO); Department of Chemistry, Aarhus University, Gustav Wieds Vej 14, DK-8000 Aarhus C, Denmark

## Abstract

The crystalline two-dimensional thiostannate Sn_3_S_7_(trenH)_2_ [tren = tris(2-aminoethyl)amine] consists of negatively charged (Sn_3_S_7_^2−^)_*n*_ polymeric sheets with trenH^+^ molecular species embedded in-between. The semiconducting compound is a violet light absorber with a band gap of 3.0 eV. In this study the compound was synthesized and functionalized by introducing the cationic dyes Methylene Blue (MB) or Safranin T (ST) into the crystal structure by ion exchange. Dye capacities up to approximately 45 mg/g were obtained, leading to major changes of the light absorption properties of the dye stained material. Light absorption was observed in the entire visible light region from red to violet, the red light absorption becoming more substantial with increasing dye content. The ion exchange reaction was followed in detail by variation of solvent, temperature and dye concentration. Time-resolved studies show that the ion exchange follows pseudo-second order kinetics and a Langmuir adsorption mechanism. The pristine and dye stained compounds were characterized by powder X-ray diffraction and scanning electron microscopy revealing that the honeycomb hexagonal pore structure of the host material was maintained by performing the ion exchange in the polar organic solvent acetonitrile, while reactions in water caused a break-down of the long-range ordered structure.

Metal oxides and -chalcogenides have been studied extensively due to their semiconducting and photocatalytic properties. With an ever increasing global demand for energy, photocatalysis has the potential to provide a sustainable solution by converting two inexhaustible resources, namely sunlight and water, into chemical fuel in the form of hydrogen^1^,^2^,^3^. Another application of photocatalysis is the degradation of organic pollutants including halogenated benzene and phenol derivatives^4^,^5^ as well as organic dyes^6^. Materials such as TiO_2_, ZnO and SnS_2_ have been employed in the photogenerated production of hydrogen^7^,^8^ and for degradation of pollutants^9^,^10^. However, the efficiencies of single phase semiconductors are often limited by charge recombination^11^,^12^, and in some cases by large band gaps. While UV-light corresponds to less than 5% of the solar spectrum^13^, materials may be functionalized to red-shift their solar light response.

Layered metal chalcogenides is a promising group of semiconducting materials with structural features that may be tuned towards desired properties such as heavy metal uptake^14^,^15^, catalysis^16^,^17^,^18^ and photocatalysis^19^,^20^. A family of violet light absorbing semiconducting two-dimensional thiostannates was denoted R-SnS-1 by Bedard and co-workers^21^. These compounds have the composition R_2_Sn_3_S_7_ (R is a monovalent cation) and consist of polymeric anionic (Sn_3_S_7_^2−^)_*n*_ sheets separated by charge stabilizing species such as alkali- or alkyl-ammonium based cations. The R-SnS-1 compounds are often prepared by templated hydrothermal or solvothermal synthesis, and the template acts as a structure directing agent in addition to being incorporated into the structure as the cation R. Templates of various nature have been used including Cs^+^ 22, Rb^+^ 23, tetramethylammonium (TMA)^24^, protonated quinuclidine (QUINH)^25^, and protonated 1,4-diazabicyclo[2.2.2]octane (DABCOH)^26^, and even metal-organic complexes such as Fe[(1,10-phenanthroline)_3_]^2+^ have been incorporated in the selenium analogues R-SnSe-1^27^.

The (Sn_3_S_7_^2−^)_*n*_ thiostannate layers are composed of Sn_3_S_4_ broken-cube clusters held together by double bridge Sn-(μ-S)_2_-Sn covalent bonds. This creates a honeycomb-like structure with 24-atom hexagonal pores with a size of approximately 1 nm. Despite consisting of the same structural motifs, the pores can be either regular or distorted hexagonal depending on the synthesis template. As the (Sn_3_S_7_^2−^)_*n*_ layers and the cationic species are only held together by electrostatic interactions, the interlayer distance is flexible and depends on the cation size. This enables post-synthetic exchange of the intercalated cations. Functionalization by ion exchange has been widely applied for layered materials including layered double hydroxides (LDHs)^28^ and three-dimensional compounds such as metal-organic frameworks (MOFs)^29^. Comparatively little has still been done in this area for R-SnS-1 type compounds, where the main focus has been on ion exchange by alkali-, alkali earth- or transition metal ions^24^,^30^,^31^,^32^. Furthermore, it has been proven that the organic cation tert-butyl ammonium (TBA) can be replaced by tetramethylammonium (TMA) while keeping the framework intact^25^.

In this paper, we report the solvothermal synthesis of lamellar Sn_3_S_7_(trenH)_2_ (tren = tris(2-aminoethyl)amine) and its subsequent functionalization by cationic organic dyes. The aim is to modify the light absorption properties of the pristine violet light absorbing material by post-synthetic modification. The intercalation of Methylene Blue (MB) and Safranin T (ST) into the thiostannate host was carried out by ion exchange in solution and studied in detail. The solvent, temperature, nature of the dye, and dye concentration were varied, and the kinetics of the reactions were investigated. The structural features of the Sn_3_S_7_(trenH)_2_-dye nanocomposites were examined by powder X-ray diffraction (PXRD) and scanning electron microscopy (SEM), while the optical properties were investigated by diffuse reflectance spectroscopy (DRS). Solid state NMR spectroscopy was used to investigate the nature of the cationic species of the parent compound Sn_3_S_7_(trenH)_2_.

## Results and Discussion

### Structural characterization of pristine Sn_3_S_7_(trenH)_2_

**Figure 1:**
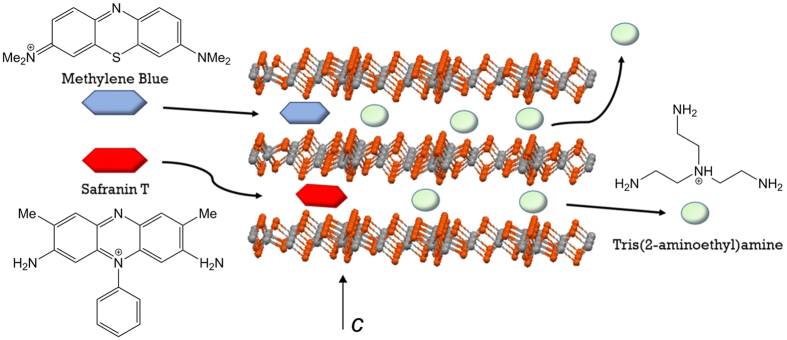
The structure of Sn_3_S_7_(trenH)_2_ consists of honeycomb (Sn_3_S_7_^2−^)_*n*_ layers with cationic species embedded in-between. Sulfur and tin atoms are displayed in orange and grey, respectively. Tris(2-aminoethyl) amine (tren) was used as the synthesis template, and trenH^+^ ions were intercalated in the structure of the as-synthesized material. The organic dyes Methylene Blue (MB) or Safranin T (ST) were introduced into the structure by ion exchange.

R-SnS-1 type compounds are stacked materials consisting of polymeric anionic (Sn_3_S_7_^2−^)_*n*_ thiostannate layers with charge stabilizing molecular cations located in-between (Fig. 1). The thiostannate layers in Sn_3_S_7_(trenH)_2_ may be described in the hexagonal space group P6_3_/*mmc* with unit cell dimensions of *a* = 13.3 Å and *c* = 19.1 Å^33^. The thiostannate layers stack in an ABAB sequence along the *c*-axis. The diameter of the hexagonal pores is 11 Å, while the interlayer distance is 9.6 Å, corresponding to half of the *c*-axis.

**Figure 2:**
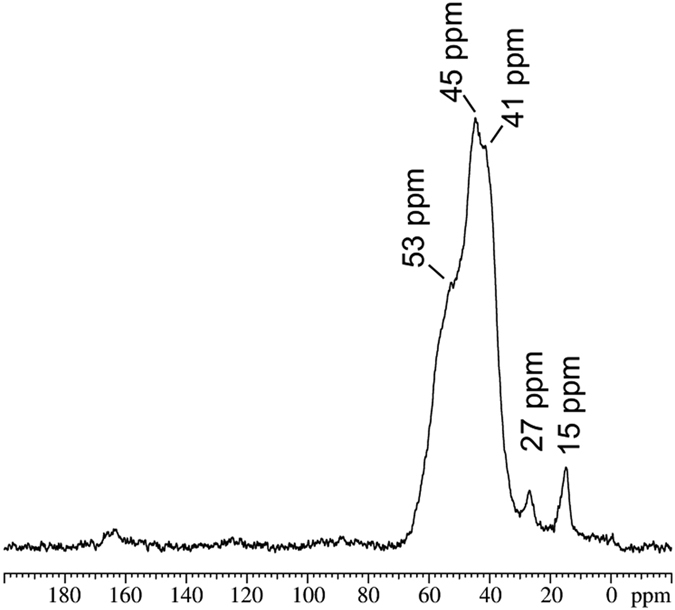
^13^C CP{^1^H}/MAS NMR spectrum (9.4 T, 4.0 kHz spinning speed) of pristine Sn_3_S_7_(trenH)_2_.

The molecular cations are statically disordered in the crystal structure of Sn_3_S_7_(trenH)_2_^33^, and the nature of the embedded cationic species has not been verified in previous studies. It is proposed by Pienack *et al*. that the molecular cations are present as protonated tren (trenH^+^) in agreement with elemental analysis^33^. In the synthesis of other R-SnS-1 type materials, however, it was reported that the synthesis template may undergo intramolecular structural changes during the solvothermal reaction^34^. In order to investigate the nature of the templating cation, a solid-state ^13^C{^1^H} CP/MAS NMR spectrum was acquired for a sample of as-synthesized Sn_3_S_7_(trenH)_2_ (Fig. 2). The spectrum shows sharp peaks of low relative intensity at 15 and 27 ppm whereas a broad peak of overlapping resonances at approximately 41, 45, 53 and 57 ppm dominates the spectrum. If trenH^+^ ions were present in an isotropic environment with a proton at the tertiary amine, the three alkyl chains would be equivalent, and only two ^13^C chemical shifts would be expected as confirmed by the theoretical chemical shifts for trenH^+^ of 38 and 44 ppm. For comparison the ^13^C chemical shifts for tren in the liquid state are 40 and 58 ppm (Fig. S1). However, the fact that the cation is disordered in the crystal structure points to small variations in the local chemical environment due to different cation-anion interactions. This results in chemical shift dispersion and thereby broad peaks. The chemical shifts of the broad peak (41–57 ppm) can be assigned to aliphatic C-N carbon atoms. The shifts at 15 and 27 ppm could represent methyl- or methylene groups, respectively. As no other solvents or carbon containing precursors than tren and acetonitrile were used for the synthesis, the peaks at low chemical shifts point to partial breakdown of tren. Due to the low intensity of these peaks in comparison with those at 41–57 ppm, we can conclude that tren is most likely present as trenH^+^ in the structure.

### Structural characterization of dye modified Sn_3_S_7_(trenH)_2_

The disorder of the molecular cations points to weak cation-anion interactions in Sn_3_S_7_(trenH)_2_ enabling ion exchange of colorless trenH^+^ by optically active cationic species. Aiming to modify the optical properties of the material, Sn_3_S_7_(trenH)_2_ was functionalized by the cationic dyes Methylene Blue (MB) and Safranin T (ST). Samples of pristine Sn_3_S_7_(trenH)_2_ were treated with commercial chloride salts of the cationic dyes. Figure 1 shows a schematic representation of the ion exchange procedure. Initial inspection of the dye stained compounds by optical microscopy (Fig. S2) suggests the dye diffuses into the crystals from the side (*i.e*. parallel to the [001] crystal faces), as illustrated in Fig. 1. Alternatively, it could have diffused through the hexagonal pores.

**Figure 3:**
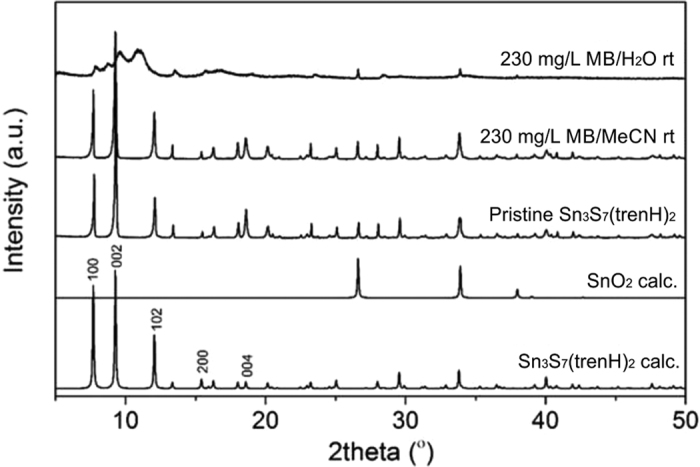
Powder X-ray diffraction data collected on a pristine and two MB stained samples in water and acetonitrile revealing that the crystal structure is maintained by performing the ion exchange in acetonitrile. Reference diagrams for SnO_2_ (ICSD 160667) and Sn_3_S_7_(trenH)_2_^33^ are shown revealing presence of a SnO_2_ impurity. Characteristic Sn_3_S_7_(trenH)_2_ peaks have been indexed.

Powder X-ray diffraction (PXRD) data were collected on pristine and dye-modified Sn_3_S_7_(trenH)_2_ (Fig. 3). The pattern for the pristine phase matches the phase reported in the literature^33^. In addition, an impurity of the SnO_2_ precursor is present; the concentration of SnO_2_ in a representative Sn_3_S_7_(trenH)_2_ sample was estimated to be 8 wt% by Rietveld refinement (Fig. S4 and Table S1). A later optimization of the synthesis has proven that phase pure Sn_3_S_7_(trenH)_2_ can be obtained by using an excess of sulfur in the solvothermal reaction (Methods Section and Fig. S5). It is expected that SnO_2_ does not affect the dye adsorption due to its chemically inert and non-porous nature (see Supplementary Information). The crystal structure of Sn_3_S_7_(trenH)_2_ treated with dye in acetonitrile (MeCN) is well preserved. In contrast, treatment of the powder with an aqueous dye solution breaks down the crystalline nature of the material leaving a blue amorphous powder in which only the SnO_2_ trace is left crystalline. This reveals a loss of long-range crystalline order of the R-SnS-1 compound.

In order to investigate the effect on the unit cell of the ion exchange, powder diffraction data were modelled by Le Bail fitting using the unit cell of pristine Sn_3_S_7_(trenH)_2_ as starting model^33^. The unit cell *c*-axis decreases with the dye content, revealing that the distance between the thiostannate layers decreases upon dye exchange (Table 1 and Fig. S3). Due to the planar nature of the MB molecule it seems plausible that the average distance between thiostannate sheets decreases when bulky species such as trenH^+^ are being exchanged. While the orientation of MB cations between the layers is unknown, they will arrange to have strong cation-anion interactions. This will be achieved by minimizing the cation-anion distance *i.e*. if the cation is parallel to the thiostannate layers. The width (Hw) of the (004) reflection increases by introducing MB (Table 1 and Fig. S3). This is either an indication of: (1) Particle size decrease along the *c*-axis, (2) increased strain in the structure or (3) presence of a distribution of interplanar distances in the sample. The two latter are very like to be the case, as MB only partly replaces trenH^+^ in the as-synthesized sample (see the quantification in the following section). For comparison, the *a*-axis only changes slightly as does the width of the (200) reflection. This shows that the thiostannate layers maintain their geometry during ion exchange. Overall, the changes of the (00 *l*) peak width and of the unit cell *c*-axis reveal ion exchange inside the crystal and not only at the crystal surfaces in agreement with optical microscopy (Fig. S2).

**Figure 4:**
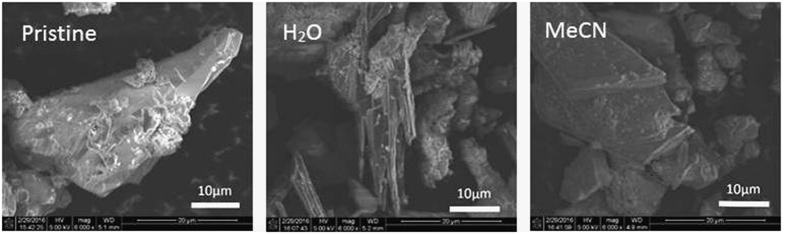
SEM images of pristine Sn_3_S_7_(trenH)_2_ and MB stained Sn_3_S_7_(trenH)_2_ functionalized in water and acetonitrile.

The structural observations are in excellent agreement with the SEM images of pristine and dye-stained Sn_3_S_7_(trenH)_2_ treated in acetonitrile and aqueous solution (Fig. 4 and Figs S6–9). Images of the pristine compound exhibit plate-like structures, owing to the microporous (Sn_3_S_7_^2−^)_*n*_ sheets spanning the unit cell *ab*-plane. SEM images of Sn_3_S_7_(trenH)_2_-dye composites treated in acetonitrile reveal the same plate morphology as the unmodified phase, which suggests that the dispersion of Sn_3_S_7_(trenH)_2_ in acetonitrile does not affect the crystal structure of the parent phase as proven by PXRD. However, treatment of the parent thiostannate with an aqueous dye solution results in needle-like domains, which were proven to be amorphous by diffraction (Fig. 3). Treatment of the pristine powder in water without a dye caused a similar loss in crystallinity, which is therefore neither related to the cation nature nor the dye concentration. Possible explanations for the structural breakdown could be either: (1) Water reacts with trenH^+^ in acid-base reactions, or (2) water acts as a nucleophile and cleaves the Sn-S bonds. The first suggestion is most likely, when taking into account that other R-SnS-1 type compounds templated by tetraalkyl ammonium ions (R_4_N^+^) may be synthesized in water^21^. The R_4_N^+^ ion will not undergo acid-base reactions with water. In contrast, modification of the charge of trenH^+^ following acid-base reactions could lead to a structural collapse due to changed cation-anion interactions between the cations and the thiostannate host. This could possibly lead to cleavage of the layers perpendicular to the stacking direction. Such cleavage would most likely result in thin disc shaped particles. Therefore, it is surprising that needle shaped domains form; however, it cannot be ruled out that the particles are plates seen from the side rather than needles. Regardless, the well-defined needles lack crystalline order. Furthermore, the argument on the acid-base reaction also conflicts the findings by C. Bowes *et al*.^25^ who demonstrated aqueous ion exchange of *tert*-butylammonium (TBA) by tetramethylammonium (TMA). They observed the framework stayed intact even though TBA may react in acid-base reactions similarly to trenH^+^. This suggests that a complex mechanism causes the breakdown of the lamellar crystal structure, and it is beyond the scope of this study to investigate this reaction in larger detail.

### Time resolved ion exchange: Effect of dye and solvent

**Figure 5:**
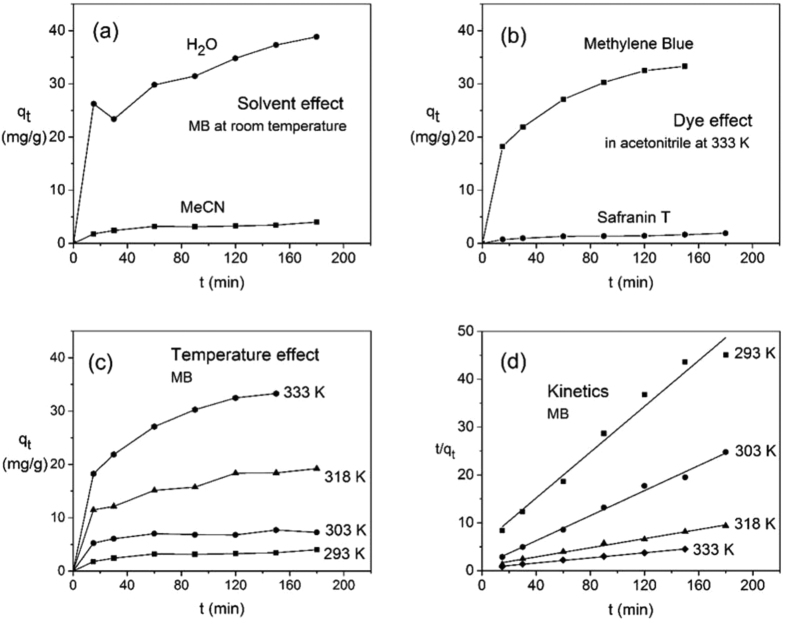
Adsorbed dye amounts *q*_t_ (in mg dye ions removed from the solution per g of adsorbent material) in Sn_3_S_7_(trenH)_2_ at different conditions using an initial dye concentration of approx. 230 mg L^−1^: (**a**) Solvent effect: Adsorbed MB at room temperature in water and acetonitrile, (**b**) Dye effect: Adsorbed MB and ST in acetonitrile at 333 K, (**c**) Temperature effect: Adsorbed MB in acetonitrile at variable temperatures. (**d**) Kinetics: Pseudo-second order kinetics model fitted to data based on (**c**).

The dye uptake is plotted as a function of reaction time in Fig. 5, as determined by UV-VIS spectroscopy (see the Methods section and the Supplementary Information). The amount of MB that is adsorbed by Sn_3_S_7_(trenH)_2_ at room temperature is substantially larger when the ion exchange is performed in water in comparison with acetonitrile (Fig. 5a) despite the structural change of the parent structure. After 3 hours at room temperature and an initial dye concentration of approx. 230 mg L^−1^, the uptake of MB reaches 38.8 mg g^−1^ from water and 4.0 mg g^−1^ from acetonitrile. This corresponds to a molar substitution of 6.0% and 0.6%, respectively, of trenH^+^ in Sn_3_S_7_(trenH)_2_, assuming that all cations have a charge of +I. Performing the adsorption in a highly polar solvent such as water most likely facilitates the ion exchange process, thereby increasing the adsorption capacity and enhancing the kinetics. Moreover, the suspected partial exfoliation of the structure in water may cause easier access to more adsorption sites. Despite the superior uptake of dye from aqueous solutions, the majority of adsorption experiments in this study are performed in acetonitrile, as to preserve the crystalline structure of the parent Sn_3_S_7_(trenH)_2_ compound.

Under similar experimental conditions, the adsorption of MB vastly exceeds that of ST (Fig. 5b). After 3 hours at 333 K in acetonitrile, the adsorption capacity just reaches 1.9 mg g^−1^ of ST (molar substitution of 0.1%) in comparison with 33.3 mg g^−1^ of MB (molar substitution of 5.1%) after 2.5 hours. This might be attributed to the pore size and interlayer distance Sn_3_S_7_(trenH)_2_, which were reported to be 10.9 × 10.9 Å and 6.04 Å, respectively, by N. Pienack *et al*.^33^ Li *et al*. have stated that adsorption can occur in micropores 1.3–1.8 times larger than the kinetic diameter of an adsorbate^35^. By using this approach, the diffusion along the hexagonal pores in Sn_3_S_7_(trenH)_2_ would allow the transport of molecules with sizes of 6.1–8.4 Å. Given the molecular dimensions of MB (14.3 × 6.1 × 4 Å^3^)^36^, it would be possible for the molecules to diffuse lengthwise through the pore channels. Similarly, the diffusion of MB molecules between the (Sn_3_S_7_^2−^)_*n*_ layers should be possible without causing changes in the interlayer distances. Despite the chemical resemblance between MB and ST (Fig. 1) the adsorption of dye onto Sn_3_S_7_(trenH)_2_ was observed to be very selective towards MB. Selectivity often arises due to either electrostatic interaction between adsorbate and adsorbent or due to structurally hindered diffusion^37^,^38^. In this case, differences in electrostatic interactions can be ruled out, as both MB and ST have charges of +I. The main structural difference between the two dyes is the additional phenyl-substituent in ST, which increases the bulkiness of ST compared to MB, especially if the phenyl ring is oriented perpendicular to the tricyclic aromatic system. The size of the dye might sterically hinder its diffusion through the pores and in-between the (Sn_3_S_7_^2−^)_*n*_ layers, thus limiting the adsorption capacity of ST in Sn_3_S_7_(trenH)_2_.

Temperature is also shown to play a significant role on the ion exchange ratio. The adsorption capacity of MB in Sn_3_S_7_(trenH)_2_ from acetonitrile at various temperatures is shown in Fig. 5c. At low temperatures, *i.e*. 293 K and 303 K, equilibrium was reached within the first 60 min., but the maximum adsorption capacities are relatively low: 4.0 mg g^−1^ at 293 K and 7.3 mg g^−1^ at 303 K. For comparison, the adsorption capacity reached 19.2 mg g^−1^ at 318 K, and 33.3 mg g^−1^ at 333 K, indicating a maximum adsorption capacity that is heavily dependent on temperature. It is not surprising that the diffusion kinetics increase with the temperature. At 333 K the ion exchange capacity is nearly as high as the room temperature ion exchange in water (Fig. 5a).

The ion exchange capacity of MB reaches an equilibrium value of 33.3 mg g^−1^ at 333 K in acetonitrile. This value is similar to adsorption of MB in *e.g*. the unfunctionalized clay mineral kaolinite (56.5 mg g^−1^ at 333 K)^39^. However, this value is low in comparison with the adsorption capacities of for example commercial activated carbon where values up to 980 mg g^−1^ have been reported^40^. However, it should be stressed that the main purpose of this study is functionalization of a smart semiconductor rather than removal of organic substances from *e.g*. waste. A member of the R-SnS-1 family templated by Me_2_NH_2_^+^ and Me_3_NH^+^ has shown Cs^+^ and Sr^2+^ exchange capacities of 409 mg g^−1^ and 65 mg g^−1^, respectively, at 338 K^41^. The latter is similar to what we observed for MB, even though the molar exchange ratio is higher for Sr^2+^ when the molar mass difference between MB and Sr^2+^ is taken into account. The higher molar exchange ratio for Cs^+^ and Sr^2+^ may be explained by the smaller size and possibly a higher diffusivity of the monoatomic cations in comparison with the cationic dyes. Importantly, the ion exchange capacity is also highly dependent on the initial dye concentration as discussed below.

### Ion exchange kinetics

The pseudo first order (PFO), pseudo second order (PSO), and intraparticle diffusion (IPD) kinetic models (see Methods section and Fig. S22) were fitted to the time resolved adsorption data for experiments carried out in acetonitrile at variable temperatures (Fig. 5c). All kinetic parameters were extracted and have been summarized in Table 2 alongside the linear correlation coefficients (*R*^2^).

Amongst the examined models, the PSO model describes the experimental data best, *i.e. R*^2^ > 0.97 at all temperatures, and reasonable agreement between calculated and experimental *q*_*e*_ values is observed (Fig. 5d and Table 2). Hence, this study suggests that the adsorption of MB onto Sn_3_S_7_(trenH)_2_ is best represented by the PSO model. A ~20% overestimation on the calculated adsorption capacity (*q*_e_, calc) is found for all datasets, which might imply that the equilibrium is not established within three hours. It is worth noting that at 318 K and 333 K, the IPD model also fit the data quite well, suggesting that the adsorption involves intraparticle diffusion. However, the linear regression does not pass through the origin (Table 2), meaning that intraparticle diffusion is not the rate-determining step. Several studies on the kinetics of dye adsorption onto various adsorbents ranging from clay to almond shells have been performed as reviewed by *e.g*. Yagub *et al*.; adsorption follows pseudo-second-order kinetics in all studies reported therein^42^.

### Ion exchange isotherms

**Figure 6:**
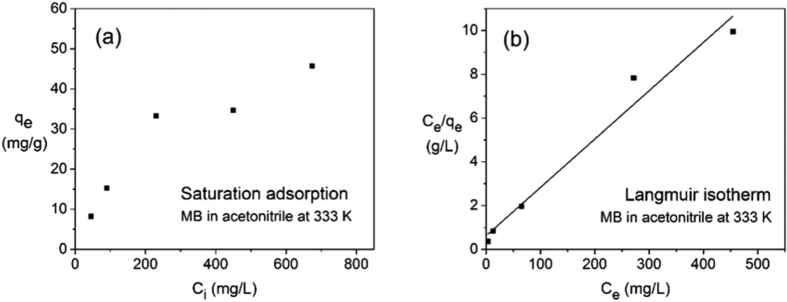
(**a**) Saturation adsorption *q*_e_ of MB at 333 K in acetonitrile as a function of the initial MB solution concentration, *C*_i_. (**b**) Langmuir isotherm model fitted to data in figure (**a**); *C*_e_ is the equilibrium dye concentration in solution.

The effect of the initial MB concentration on the equilibrium adsorption capacity was investigated using acetonitrile solutions with initial dye concentrations of 45, 90, 230, 450, and 675 mg L^−1^ as shown in Fig. 6a. The adsorption capacity increases with increasing initial dye concentration over the whole range, although at a diminishing effect at higher concentrations. Linearized Langmuir, Freundlich, and Temkin adsorption isotherms (see Methods section) were fitted to the adsorption data in Fig. 6a. The corresponding isotherm constants and correlation coefficients (*R*^2^) are summarized in Table 3 and the fits are shown in Figs 6b and S23.

Based on the correlation coefficients (*R*^2^), the model describing the experimental data best is the Langmuir isotherm. The parameter *q*_m_ represents the maximum adsorption capacity in the Langmuir model, and at a calculated value of 45(4) mg g^−1^, the model is in good agreement with the experimentally determined equilibrium adsorption capacity of 45.7 mg g^−1^ obtained from an initial concentration of 675 mg L^−1^. This suggests that the adsorbent has reached saturation, and that an increase in dye concentration would not further increase the adsorption capacity. A Langmuir isotherm model assumes a homogeneous adsorbent surface, where all sites are of equal energy for adsorption, and only a monolayer is formed. This is in good agreement with the fact that the interlayer distance and pore channel dimensions only allow for the presence of a single layer of dye molecules. Moreover, electrostatic interactions would disfavor a multilayer. The Langmuir adsorption is in good agreement with the findings by Qi *et al*. for Cs^+^ and Sr^2+^ exchange in (Me_2_NH_2_)_4/3_(Me_3_NH)_2/3_Sn_3_S_7_·1.25H_2_O^41^.

### Dye desorption tests

The potential application of the dye-stained Sn_3_S_7_(trenH)_2_ depends not only on the adsorption capacity, but also on the interactions between organic dyes and thiostannate adsorbent *i.e*. the material stability. The stability of selected dye-stained materials in solution has been investigated. Ideally, no dye should desorb by dispersing the hybrids in solution. Desorption studies of MB and ST adsorbed onto Sn_3_S_7_(trenH)_2_ were conducted in acetonitrile (see Methods section) revealing a much larger degree of desorption of ST than of MB (Figs S20–S21). From a MB adsorption capacity of 33.3 mg g^−1^, the desorption just reaches 0.15% of the amount initially adsorbed, while that of ST corresponds to 65% from the adsorption capacity of 1.9 mg g^−1^. Both experiments reach equilibrium within 60 min. Desorption is only likely to occur in the presence of other cationic species in the solution. Alternatively, it points to deposition of charge neutral species (cationic dye stabilized by a counter ion) on the crystal surface rather than ion exchange. This might be the reason for the large ST desorption, which has not been investigated in larger detail. In conclusion, the MB stained samples are stabile in solution in contrast to the ST functionalized samples.

### Optical properties of pristine and dye modified Sn_3_S_7_(trenH)_2_

**Figure 7:**
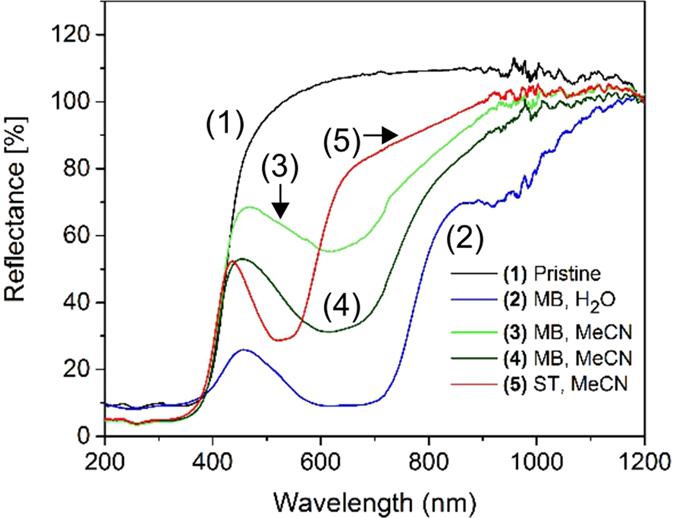
Diffuse reflectance spectroscopy data shown as a function of the wavelength. (1): pristine Sn_3_S_7_(trenH)_2_, *E*_g_ = 2.95 eV (black); (2): MB in H_2_O, 293 K, *q*_e_ = 38.8 mg g^−1^; (3): MB in MeCN, 293 K, *q*_e_ = 4.0 mg g^−1^, *E*_g_ = 3.02 eV (green); (4): MB in MeCN, 333 K, *q*_e_ = 33.3 mg g^−1^, *E*_g_ = 3.03 eV (olive); (5): ST in MeCN, 333 K, *q*_e_ = 1.9 mg g^−1^, *E*_g_ = 3.05 eV (red). The band gap of the X-ray amorphous sample (2) was not determined.

Diffuse reflectance spectroscopy (DRS) data covering the UV-VIS-nearIR range (200–1200 nm) were collected to study changes in light absorption as a consequence of dye functionalization. Spectra of pristine Sn_3_S_7_(trenH)_2_ and selected stained samples are shown in Fig. 7. The band gaps were calculated based on the reflectance data (Methods section and Fig. S24).

All samples have a bandgap of 3.0 eV/413 nm corresponding to violet light, which is consistent with the value provided for pristine Sn_3_S_7_(trenH)_2_ in the literature^33^. Samples stained with MB moreover have significant light absorption around 2 eV/620 nm (orange-red light). The ST functionalized sample also changes its visible light absorption, despite the low adsorbed equilibrium concentration. It shows significant absorption around 2.4 eV/520 nm in addition to the violet light absorption. We suggest the light absorption around 620 nm (MB)/520 nm (ST) and 413 nm correspond to excitation of the dye and the (Sn_3_S_7_^2−^)_*n*_ semiconductor, respectively. The small SnO_2_ impurity in the samples may contribute to the UV light absorption properties, hence to the potential photocatalytic properties, as SnO_2_ absorbs light around 300 nm. However, the impurity was found to have a negligible effect on the light absorption (Fig. S25).

The absorption edge at 413 nm is expected to be associated with an intralayer ligand-to-metal charge transfer (LMCT) from the S^2−^ (3p) orbital to the Sn^4+^ (5d) orbital in the (Sn_3_S_7_^2−^)_*n*_ host layers^43^,^44^. Presumably, the higher wavelength light absorption is related to the HOMO-LUMO excitation of the organic dye. More studies are needed to elucidate whether the excitation of the dye leads to charge transfer to the conduction band of the thiostannate layers, similarly to sensitizers in *e.g*. the Grätzel cell^45^. Figure S24f,g show Kubelka-Munk plots of pure MB and ST, revealing band gaps of 1.37 eV for MB and 2.08 eV for ST, respectively. The absorption energy of pure MB appears to be approximately 0.2 eV lower than the absorption energy of MB embedded inside the thiostannate. This may be explained by the fact that the excited state gets destabilized when the molecules are embedded between negatively charged layers. This leads to larger HOMO-LUMO separation, hence a higher excitation energy^46^. The effect seems less pronounced for ST, maybe due to the proposed weaker interaction between ST and the thiostannate.

Not surprisingly, a correlation was found between the amount of MB adsorbed during the adsorption experiments, and the absorption intensity in the high wavelength visible region. The more dye, the larger the orange-red light absorption. Nevertheless, there is a large difference between the samples (2) and (4), for which the saturation adsorption is similar (38.8 mg g^−1^ for MB staining in H_2_O and 33.3 mg g^−1^ for MB staining in MeCN). This may be related to the fact that the water treated sample has lost its long-range order.

## Conclusion

This study is a proof-of-concept that R-SnS-1 type materials can be post-synthetically functionalized by ion exchange. Pristine Sn_3_S_7_(trenH)_2_ has been synthesized and modified by ion exchange of the organic dyes Methylene Blue and Safranin T, and detailed insight is given into the sensitization of layered semiconducting Sn_3_S_7_(trenH)_2_. The effects of solvent, temperature and dye concentration were studied in detail revealing that the ion exchange follows pseudo-second order kinetics, and the adsorption follows a Langmuir isotherm. Evidence of size-dependent adsorption of the dyes was found, restricted by the pore size and interlayer distance of the host (Sn_3_S_7_^2−^)_*n*_ layers. The distance between the thiostannate layers decreases with increasing dye loading, but the parent crystal structure remains by performing the functionalization in organic solvents such as acetonitrile.

The adsorption of dyes changed the optical properties of the material, which might pave the way for visible light-responsive Sn_3_S_7_(trenH)_2_-based photocatalysis. The pristine violet light absorber was modified to have significant orange and red light absorption. Thereby, this research may improve the design of cheap, non-toxic dye-sensitized semiconductors for light harvesting as demonstrated by the changes in the light absorption properties of the materials.

## Methods

The chemical precursors SnO_2_ (≥99.9%), sulfur (≥99.5%), tris(2-aminoethyl)amine (C_6_H_18_N_4_, tren, 96%), Methylene Blue (MB) chloride, Safranin T (ST) chloride and all solvents were obtained from Sigma-Aldrich. All reagents were used without further purification. As the water content of the salts was not taken into account in the dye adsorption studies, the absolute dye concentrations may be lower than the reported values. The same precursors were used for all experiments which are therefore comparable.

### Synthesis of Sn_3_S_7_(trenH)_2_

The Sn_3_S_7_(trenH)_2_ samples used for dye adsorption studies were prepared using a solvothermal method: A mixture of SnO_2_ and sulfur (Sn:S = 3:7) was stirred in tren under ambient conditions for approximately 20 min. The solution was transferred to a Teflon-lined stainless steel autoclave for thermal treatment at 190 °C for 6 days. The resulting pale green-yellow crystals were washed with acetonitrile and subsequently air-dried for at least 24 hours. The samples were found to contain an impurity of SnO_2_ (Fig. 3). A later synthesis of Sn_3_S_7_(trenH)_2_ was carried out using the same procedure but with a molar ratio of Sn:S = 3:8. This resulted in a phase pure product (Fig. S5).

### Powder X-ray diffraction (PXRD)

Powder X-ray diffraction was performed using a SmartLab diffractometer from Rigaku equipped with a Cu source. Cu*Kα*_1_ radiation (*λ* = 1.54056 Å) was used to collect data at room temperature on flat silicon sample holders. Le Bail fitting was performed to extract unit cell parameters and zero-point corrected peak positions from the diffraction data using the FullProf Suite^47^. The same program was used to fit pseudo-Voigt functions to selected reflections to determine the width of these diffraction peaks and to quantify the SnO_2_ impurity by Rietveld refinement.

### Solid state nuclear magnetic resonance spectroscopy (NMR)

The ^13^C{^1^H} CP/MAS NMR spectrum was obtained on a Bruker Avance II 400 MHz (9.4 T) spectrometer using a home-built CP/MAS NMR probe for 7 mm outer-diameter rotors, v_R_ = 4.0 kHz, a 8-s relaxation delay, a CP contact time of 0.5 ms, and 8192 scans.

### Diffuse reflectance spectroscopy (DRS)

Diffuse reflectance spectroscopy (DRS) was used to investigate the light absorption properties in the wavelength range (200–1200 nm) using a Shimadzu UV-3600 spectrophotometer. Band gaps were calculated based on DRS data converted to the Kubelka-Munk function, where *R* is the reflectance:


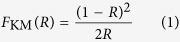


### Scanning electron microscopy (SEM)

Powders were immobilized on conducting carbon tape, and scanning electron microscopy images were acquired on a FEI-Nova NanoSEM 600 scanning electron microscope.

### Dye adsorption/ion exchange protocol and quantification

All dye-adsorption experiments were carried out by suspending 0.2 g of Sn_3_S_7_(trenH)_2_ in a 40.0 mL dye-solution and stirring for 3–4 hours in a sealed Erlenmeyer flask. Experiments were performed using different organic dyes (MB and ST), initial dye-concentrations (MB^+^ or ST^+^: 45–675 mg L^−1^), temperatures (293–333 K), and solvents (water and acetonitrile). The resulting dye concentrations were determined by UV-VIS absorption spectroscopy at the maximum absorbance wavelength (*i.e*. 665 nm for MB in H_2_O, 655 nm for MB in acetonitrile, and 518 nm for ST in acetonitrile). A Shimadzu UV-1800 spectrophotometer was used for these measurements. The amount of dye adsorbed by the thiostannate, *q*_*t*_ (mg g^−1^), was calculated according to the following equation:


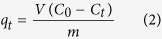


where *V* is the solution volume (L), *C*_0_ is the initial dye concentration (mg L^−1^), *C*_*t*_ is the concentration at contact time *t* (mg L^−1^), and *m* is the mass of adsorbent (g). As the adsorption reaches equilibrium, the above equation becomes:


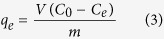


where *q*_*e*_ is the equilibrium adsorption capacity (mg g^−1^), and *C*_*e*_ is the equilibrium dye concentration in the solution (mg L^−1^).

### Adsorption kinetics modelling

For each adsorption experiment a series of measurements were conducted at various reaction times. In order to interpret the results, different kinetic models were fitted to data: The pseudo-first order (PFO)^48^, pseudo-second order (PSO)^49^, and intraparticle diffusion (IPD)^50^ models. The linearized forms of these models are as expressed below:


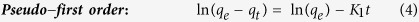



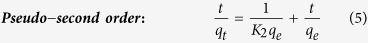



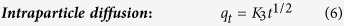


In these equations *q*_*t*_ and *q*_*e*_ have the same definitions as in Eq. 2 and Eq.3, *t* is contact time, while *K*_1_ (min^−1^), *K*_2_ (g mg^−1^ min^−1^), and *K*_3_ (mg g^−1^ min^−0.5^) are the rate constants of the PFO, PSO, and IPD model, respectively. As the intraparticle model fits do not intersect the origin a constant *C* was introduced in the fits: *q*_*t*_ = *K*_3_*t*^1/2^ + *C*.

To compare the adsorption capacity predicted by the models to that found in the experiments, a percent deviation (Δ*q*) was calculated as follows:


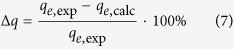


where *q*_*e*,exp_ and *q*_*e*,calc_ are the experimental and calculated equilibrium adsorption capacity, respectively.

### Equilibrium isotherm modeling

The relationship between the adsorption capacity at equilibrium and the amount of adsorbate (dye) left in solution is important in describing the interaction between adsorbent and adsorbate. Several isotherm models are available^51^,^52^,^53^,^54^,^55^,^56^, and fitting the isotherm equations to equilibrium data can provide insight into the adsorption mechanism. In this paper the Langmuir, Freundlich, and Temkin isotherm models have been fitted to the experimental data.

#### Langmuir isotherm

The Langmuir model assumes a homogenous adsorbent surface, *i.e*. all sites are of equal energy for adsorption, and only a monolayer coverage of these sites is possible^53^. The model is defined by the following equation:


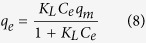


In a linear form (eq. 8) is presented as:


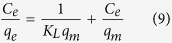


where *q*_*e*_ is the equilibrium adsorption capacity (mg g^−1^), *C*_*e*_ is the equilibrium adsorbate concentration in solution (mg L^−1^), *K*_*L*_ is the Langmuir constant (mg L^−1^), and *q*_*m*_ is the maximum adsorption capacity for a monolayer coverage (mg g^−1^).

#### Freundlich isotherm

The Freundlich model describes a heterogeneous adsorbent surface, where the adsorption energy is not equal for all sites^55^. The Freundlich equation is as expressed below:


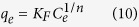


This can be written in the linear form:


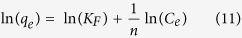


where *K*_*F*_ and *n* are constants indicating adsorption capacity (mg g^−1^) and favorableness (unit-less), respectively. The constant *n* is usually interpreted as a measure of surface heterogeneity; its value ranging between 1 and 10, the lower the value the more heterogeneous the surface.

#### Temkin isotherm

The Temkin model is based on the Langmuir model, but takes into account indirect adsorbate-adsorbate interactions at the adsorption sites. The model also assumes that the heat of adsorption will decrease linearly with surface coverage^56^. The model is expressed by the following equation:


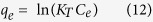


which in a linear form is presented as:


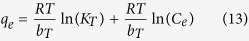


where *R* is the gas constant (8.314 J mol^−1^ K^−1^), *T* is absolute temperature (K), while *K*_*T*_ is the Temkin constant corresponding to maximum binding energy (L g^−1^), and *b*_*T*_ is related to the heat of adsorption (J mol^−1^).

### Dye desorption protocol and quantification

The stability of the Sn_3_S_7_(trenH)_2_-organic dye hybrids in solution was analyzed in desorption experiments. Selected dye-stained products were suspended in acetonitrile, and the amount of desorbed dye was determined by UV-Vis absorption spectroscopy. The percentage of desorption was calculated by the following expression:


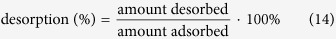


## Additional Information

**How to cite this article:** Hvid, M. S. *et al*. Light absorption engineering of a hybrid (Sn_3_S_7_^2-^)*_n_* based semiconductor – from violet to red light absorption. *Sci. Rep.* **7**, 45822; doi: 10.1038/srep45822 (2017).

**Publisher's note:** Springer Nature remains neutral with regard to jurisdictional claims in published maps and institutional affiliations.

## Supplementary Material

Supplementary Information

## Figures and Tables

**Table 1:** Unit cell data determined by Le Bail fitting of PXRD data reveal that the *a*-axis largely remains unchanged by ion exchange, while introduction of MB decreases the *c*-axis length representing the interlayer distance.

Sample	*a* (Å)	*c* (Å)	2*θ* (200) (degrees)	Hw (200) (degrees)	2*θ* (004) (degrees)	Hw (400) (degrees)
Pristine	13.2474 (4)	19.0661 (8)	15.434	0.0641 (8)	18.600	0.1557 (3)
230 mg g^−1^ MB/MeCN (RT)	13.2403 (4)	19.003 (1)	15.442	0.0621 (7)	18.662	0.2123 (6)
450 mg g^−1^ MB/MeCN (333 K)	13.2169 (6)	18.868 (1)	15.470	0.070 (4)	18.797	0.211 (1)
675 mg g^−1^ MB/MeCN (333 K)	13.2301 (5)	18.853 (1)	15.454	0.063 (4)	18.812	0.221 (1)

Introduction of MB into the structure leads to an increase in the (004) peak width, while the (200) width remains unchanged. The (004) rather than the more intense (002) reflection is reported as the (002) and (101) reflections partly overlap. The dye concentrations refer to the concentration of the cationic dye *i.e*. without the chloride ion. The peak widths were determined by single peak fitting.

**Table 2:**
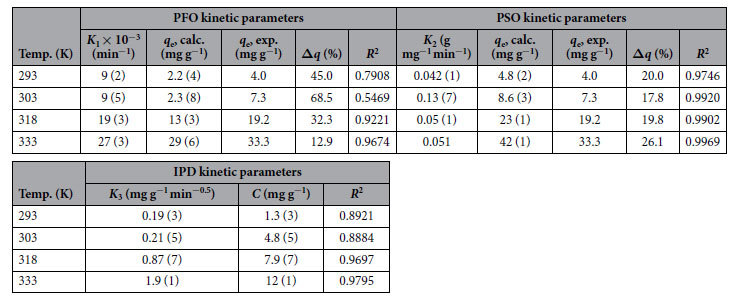
Kinetic parameters from pseudo first order (PFO), pseudo second order (PSO) and intraparticle diffusion (IPD) kinetics.

**Table 3:** Langmuir, Freundlich and Temkin isotherm data.

Langmuir		Freundlich		Temkin	
*K*_L_ (L/mg)	0.03 (3)	*K*_F_ (mg/g)	6 (1)	*K*_T_ (L/g)	1.0 (6)
*q*_m_ (mg/g)	45 (4)	*n*	3.0 (4)	*b*_T_ (J/mol)	395 (56)
*R*^2^	0.9729	*R*^2^	0.9692	*R*^2^	0.9423
