# Supplementary material for: Light absorption engineering of a hybrid (Sn3S72−)n based semiconductor – from violet to red light absorption
Source: Sci Rep. 2017 Apr 4;7:45822. doi: 10.1038/srep45822 (PMC5379188; doi:10.1038/srep45822)
Supplement: Supplementary Information [file srep45822-s1.pdf]

SUPPLEMENTARY INFORMATION: Light absorption engineering of a hybrid $(Sn_3S_7^{2-})_n$ based semiconductor – from violet to red light absorption

Mathias Salomon Hvid[1], Paolo Lamagni[2] & Nina Lock[2]*

[1]Interdisciplinary Nanoscience Center (iNANO), Aarhus University, Gustav Wieds Vej 14, DK-8000 Aarhus C, Denmark

[2]Carbon Dioxide Activation Center (CADIAC), Interdisciplinary Nanoscience Center (iNANO) and Department of Chemistry, Aarhus University, Gustav Wieds Vej 14, DK-8000 Aarhus C, Denmark

Corresponding author: nlock@chem.au.dk

This supplementary information contains figures, tables, and comments to support the explanations in the manuscript.

Solid and liquid state ^{13}C NMR

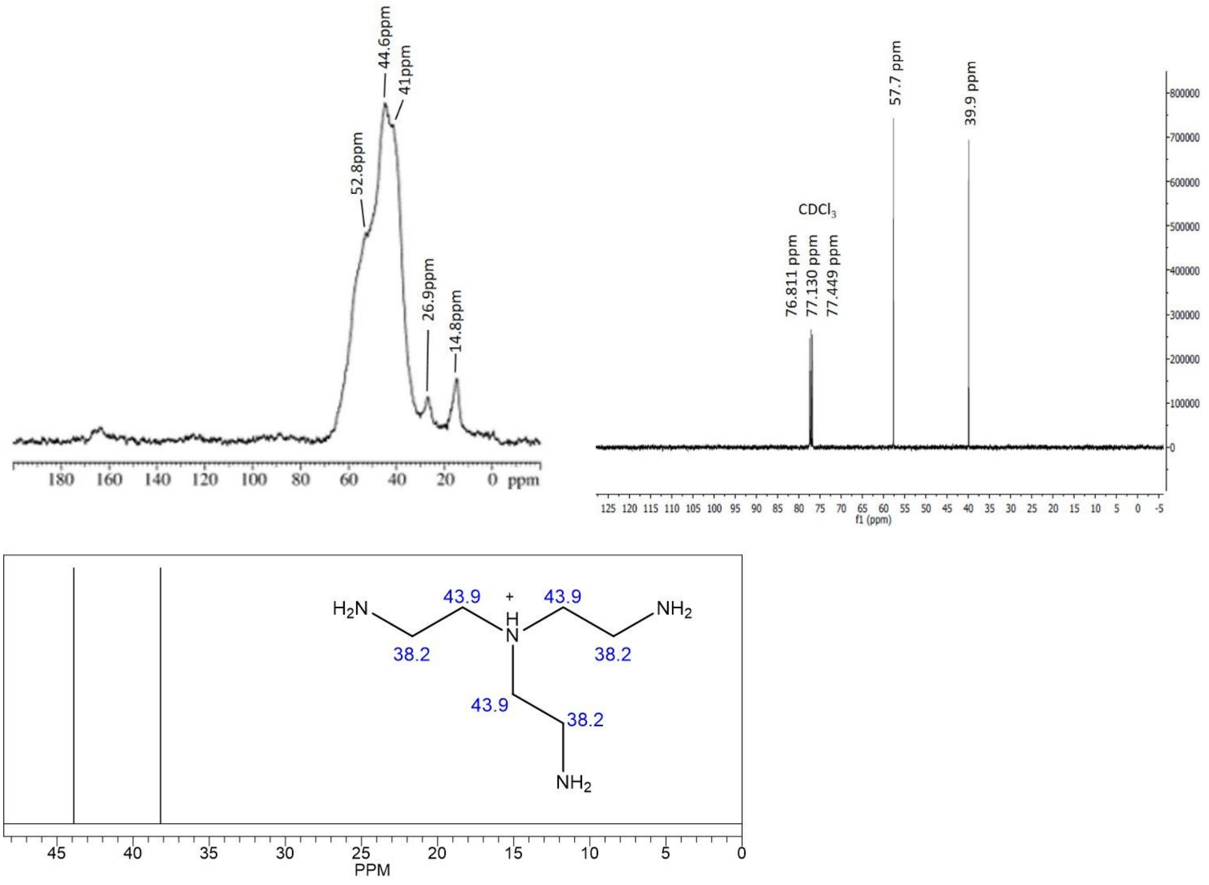

Figure S1: (*Top left*) Experimental solid state ^{13}C CP/MAS NMR spectrum of $Sn_3S_7(trenH)_2$, (*top right*) experimental liquid state ^{13}C NMR spectrum of tren in $CDCl_3$, and (*bottom*) theoretical liquid phase ^{13}C NMR chemical shifts of $trenH^+$ (*ChemDraw Professional 15.1* software). While there are several chemical shifts in the solid state spectrum due to the disordered nature of the amine within the $(Sn_3S_7^{2-})_n$ lattice, reasonable agreement was found around 40 ppm between the experimental solid state spectrum and the predicted spectra for the $trenH^+$ ions. Shifts at 14.8 ppm and 26.9 ppm can most likely be attributed to minor products of the redox chemistry taking place during the synthesis of $Sn_3S_7(trenH)_2$.

Optical microscopy images

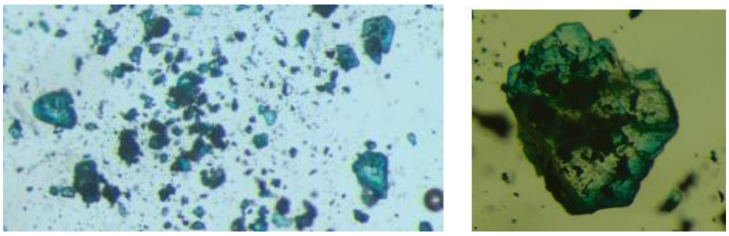

Figure S2: Optical microscopy images of Methylene Blue (MB) stained $Sn_3S_7(trenH)_2$. The short dimension of the crystalline plates corresponds to the direction of the crystallographic *c*-axis. The darker color at the crystal edges on the right hand figure suggests the ion exchange takes place by diffusion parallel to the layers rather than through the hexagonal pores perpendicular to the [001] planes.

Powder diffraction

Powder diffraction data were Le Bail fitted using a Thomson-Cox-Hastings pseudo-Voigt function to describe the peak profiles. As a spherical model was used to describe data, the profile description of the stained samples was far from ideal due to increasing broadening of the (00*l*) reflections with dye concentration. The only parameters that were extracted from the data fits were the unit cell and the zero-point corrected (200) and (004) peak positions (Figure S3).

Single peaks were fitted to determine the peak width of the (200) and (004) reflection. The (200) reflection was chosen over the (100) reflection due to large peak asymmetry of the (100) reflection, while the (004) was fitted rather than the (002) reflection which is partly overlapping with the (101) reflection.

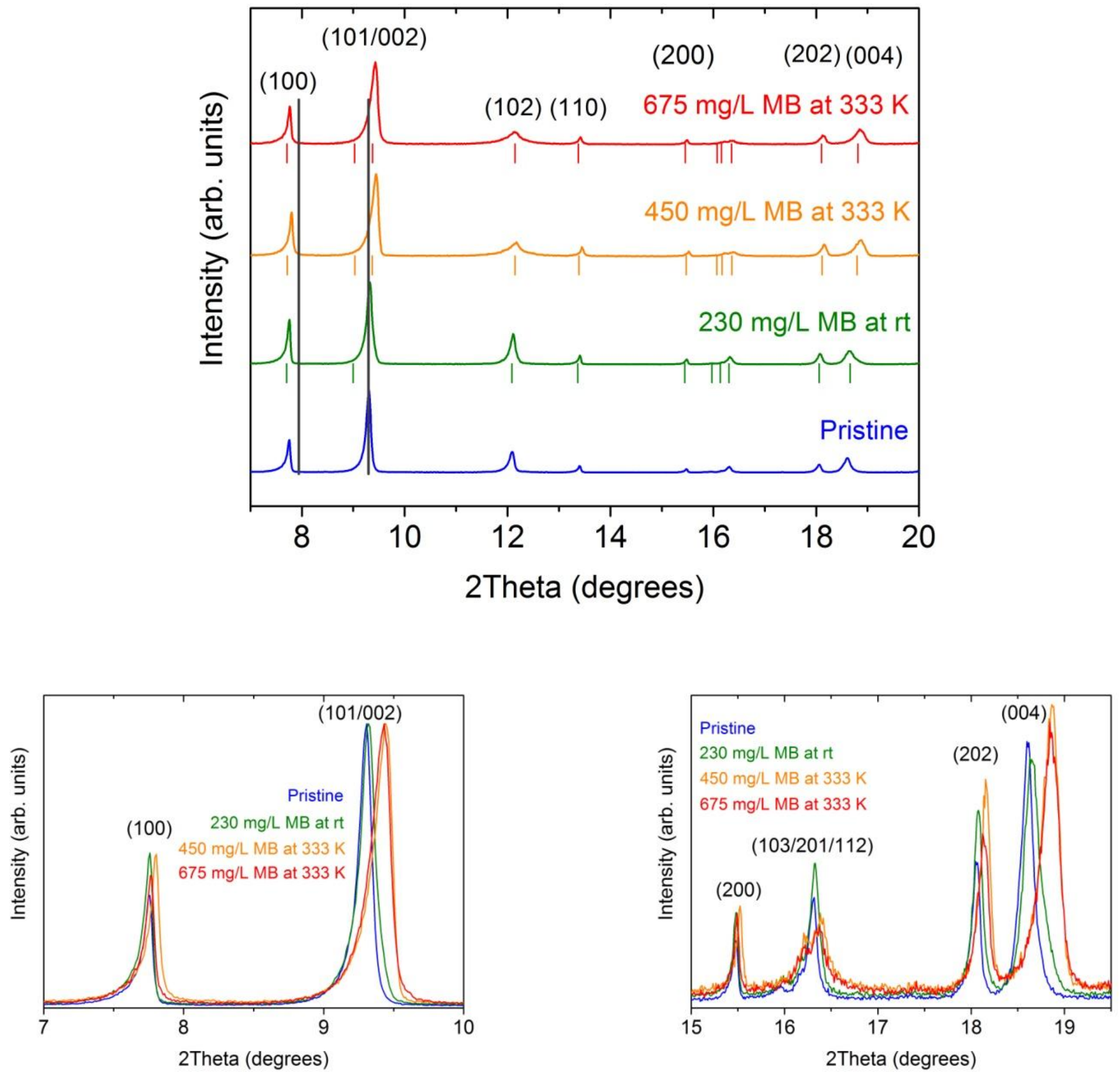

Figure S3: (*Top*) Normalized powder diffraction data are shown along with the Bragg peak positions of the refined unit cell. The vertical grey lines are guides to the eye revealing that the unit cell *c*-axes changes with the adsorbed dye amount. (*Bottom*) Representation of selected areas of normalized PXRD data revealing increasing peak width and a change in peak position of the (00*l*) reflections with increasing dye amount.

Rietveld refinement of powder X-ray diffraction data was performed to estimate the concentration of residual SnO_2 precursor in a $Sn_3S_7(trenH)_2$ sample. The diffraction data were collected on a different $Sn_3S_7(trenH)_2$ sample than the one used for dye adsorption studies shown in the manuscript Figure 3. However, as the two samples were synthesized using the same protocol, the estimates provided here were taken as a representative for all samples prepared by the same protocol.

Atomic coordinates from Filsø *et al.*[1] were used as a starting model to describe $Sn_3S_7(trenH)_2$, while ICSD entry 160667 was used as a starting model for the SnO_2 phase. The peak profiles were described by a Thomson-Cox-Hastings pseudo-Voigt function (*X* and *W*). For both phases the thermal vibration was described by an overall *B*-factor. Two parameters were used to describe the preferred orientation (*Or1* and *Or2*) using a Modified March's function, while the parameters S_L and D_L describe the peak asymmetry of $Sn_3S_7(trenH)_2$.

The results of the refinement have been summarized in Table S1 and are shown in Figure S4. The fit is far from perfect (reflected in the large *R*-factors) due to preferred orientation in the sample. Moreover, presence of disordered $trenH^+$ was not taken into account in the model. Therefore, the refined SnO_2 concentration of 8 wt% should be considered an estimate only.

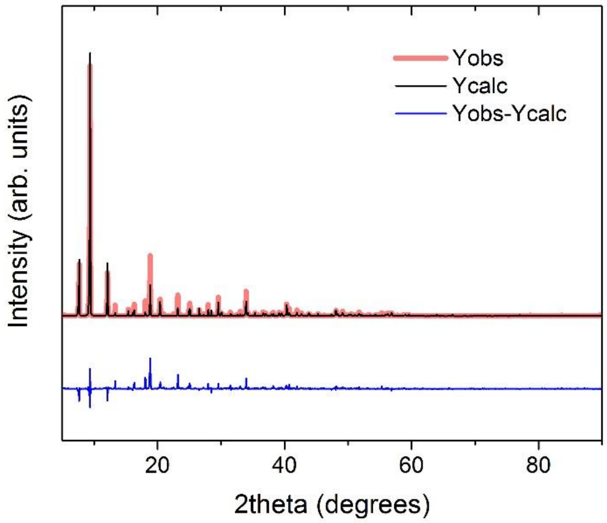

Figure S4: Powder X-ray diffraction data (red) and model (black) used to estimate the amount of SnO_2 impurities in the $Sn_3S_7(trenH)_2$ sample.

	$Sn_3S_7(trenH)_2$		SnO_2	
	Bragg R-factor (%)	23.6	Bragg R-factor (%)	17.0
	Rf-factor (%)	20.4	Rf-factor (%)	10.8
	Fraction (%)	92(1)	Fraction (%)	7.9(2)
**Refined parameters**	Scale ($\times 10^{-6}$)	3.75(3)	Scale ($\times 10^{-5}$)	1.00(3)
	Zero	0.1422(5)	Zero	0.1422(5)
	a, Å	13.2661(2)	*a*, Å	4.7406(2)
	c, Å	18.7955(3)	*c*, Å	3.1857(2)
	W	0.00289(9)		
	X	0.02136(8)		
	S_L	0.158(4)		
	D_L	0.158(4)		
	Or1	0.625(9)		
	Or2	0.55(2)		
	Overall *B*-factor ($Å^2$)*	2.2803	Overall *B*-factor ($Å^2$)*	1.5748
			*W**	0.004332
			*X**	0.18105

Table S1: Powder X-ray diffraction refinement parameters and the weight ratio estimates of the $Sn_3S_7(trenH)_2$ and SnO_2 phases. The parameters marked by asterisks were optimized and then fixed. The *c*-axis of $Sn_3S_7(trenH)_2$ is shorter than that of the pristine sample presented in the manuscript and Figure S3. Data for all samples in Figure S3 were collected and refined identically allowing direct comparison between these samples.

Optimized synthesis

By mixing the precursors in a molar ratio of Sn:S = 3:8 the phase pure thiostannate is synthesized.

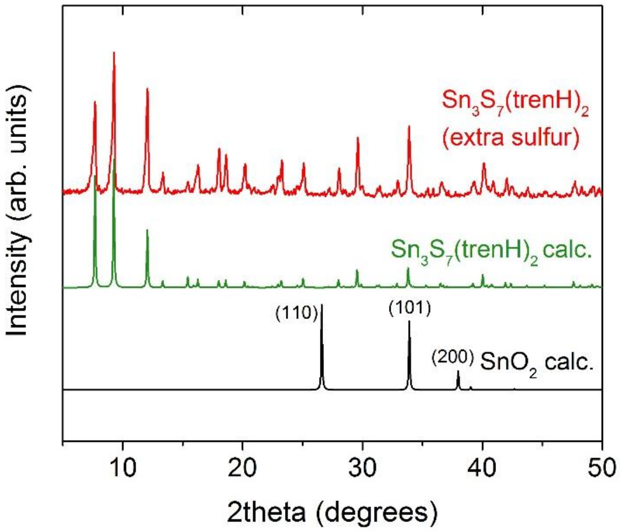

Figure S5: Powder X-ray diffraction data of the $Sn_3S_7(trenH)_2$ sample synthesized with a Sn:S molar ratio of 3:8. Reference diagrams for SnO_2 (ICSD 160667) and $Sn_3S_7(trenH)_2$[2] are shown, indicating no presence of SnO_2 impurities in the $Sn_3S_7(trenH)_2$ sample. Notice that the SnO_2 (110) reflection is not observed in the measured data, while the SnO_2 (101) reflection is overlapping with a peak from the $Sn_3S_7(trenH)_2$ phase.

Scanning electron microscopy (SEM)

Figure S6-S9 display SEM images.

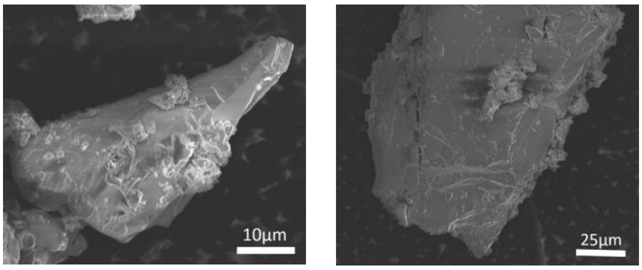

Figure S6: SEM images of pristine $Sn_3S_7(trenH)_2$.

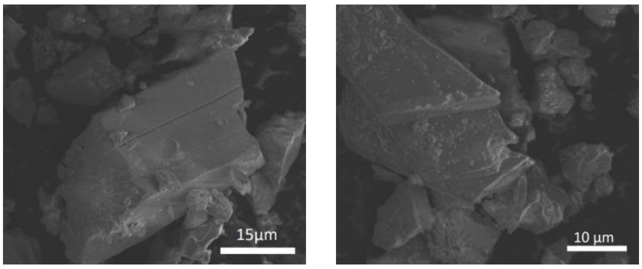

Figure S7: SEM images of samples stained with MB from 230 mg L^{-1} MeCN solution at RT.

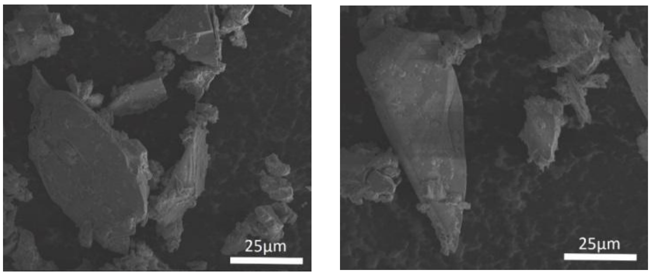

Figure S8: SEM images of samples stained with MB from 230 mg L^{-1} MeCN solution at 333 K.

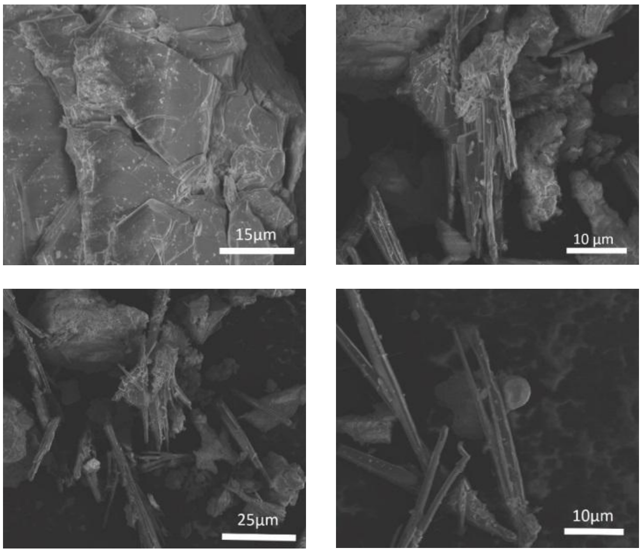

Figure S9: SEM images of samples stained with MB from 230 mg L^{-1} aqueous solution at RT.

UV-Vis spectroscopy

The adsorption of MB and ST onto $Sn_3S_7(trenH)_2$ was followed by UV-Vis spectroscopy. The spectrum at t = 0 was taken on the starting solution before the addition of $Sn_3S_7(trenH)_2$. The progress was followed as the time dependent absorbance at 665 nm (MB in H_2O), 655 nm (MB in MeCN), and 518 nm (ST in MeCN). Figure S10-S19 show UV-Vis data. Some reaction solutions were diluted prior to the UV-Vis measurements.

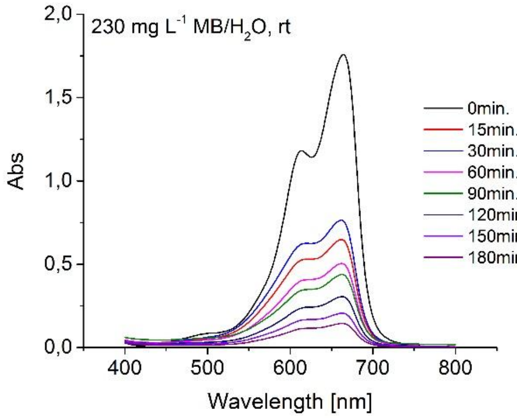

Figure S10

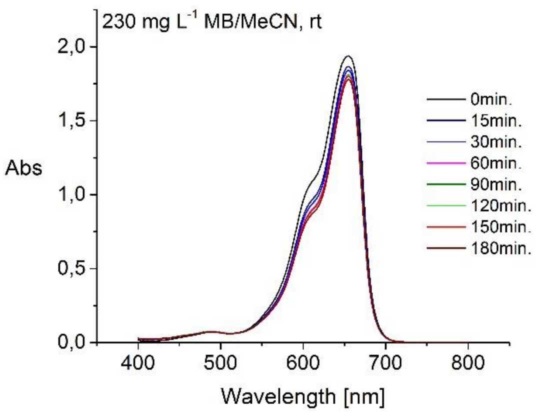

Figure S11

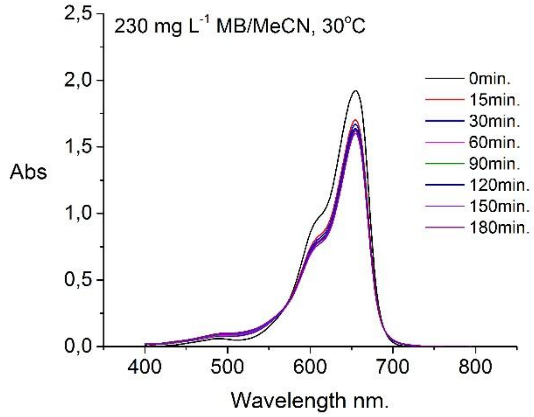

Figure S12

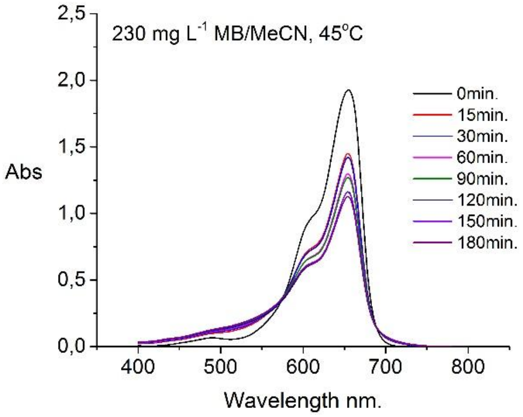

Figure S13

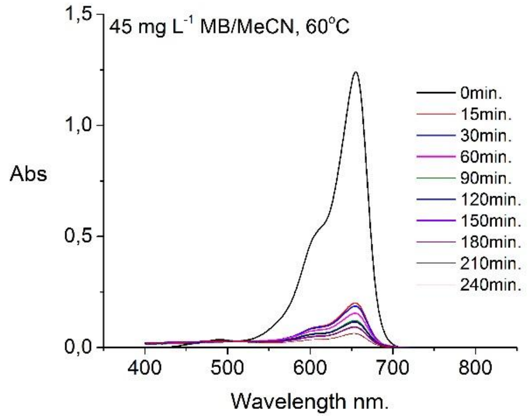

Figure S14

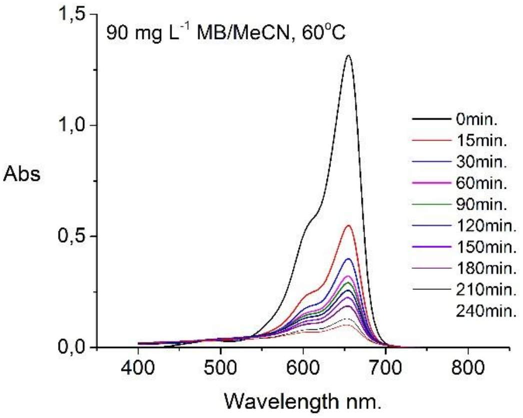

Figure S15

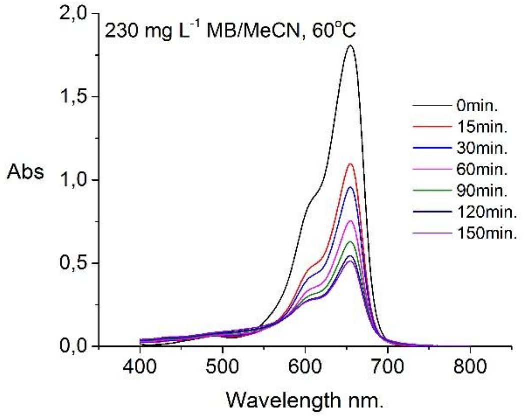

Figure S16

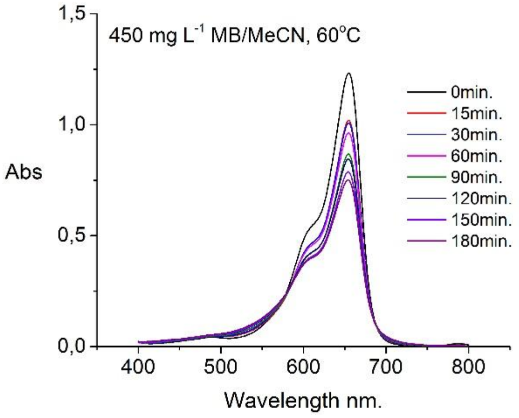

Figure S17

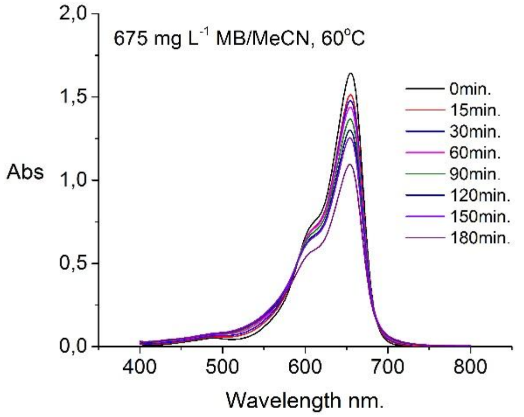

Figure S18

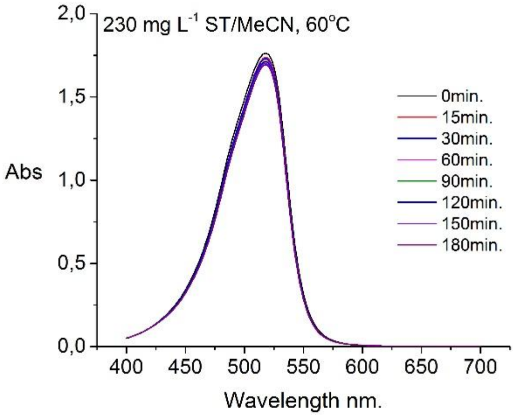

Figure S19

Dye desorption UV/Vis spectroscopy spectra

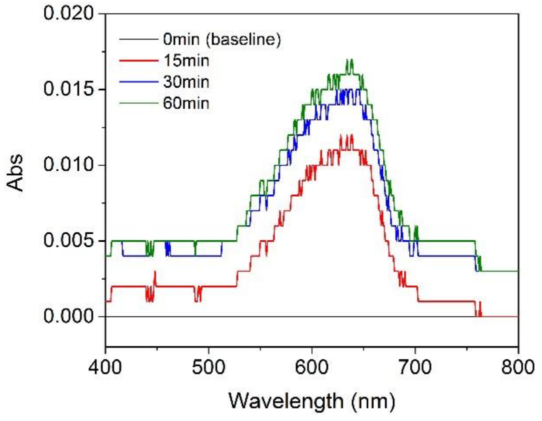

Figure S20 MB desorption.

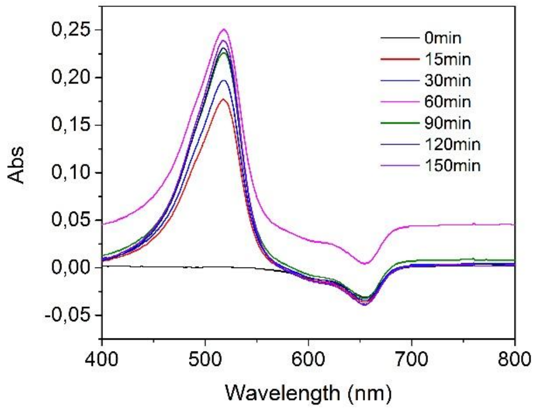

Figure S21. ST desorption.

The noise is due to the low desorbed MB concentration.

Fitting of kinetic models to MB adsorption data

Figure S22 shows plots from the kinetic modelling.

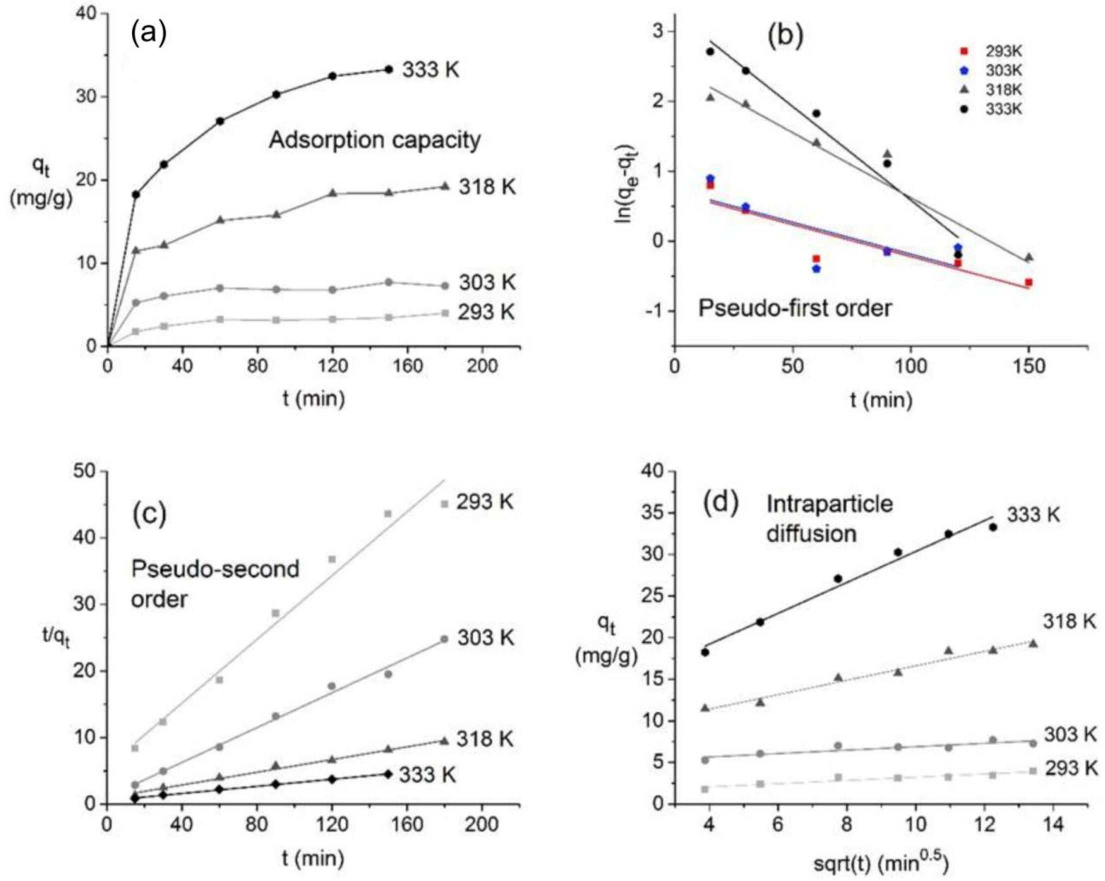

Figure S22: (a) Adsorption capacity over time at various temperatures (MB in MeCN). Kinetic models: (b) pseudo-first order, (c) pseudo-second order, (d) intraparticle diffusion.

Fitting of isotherm models to MB adsorption data

Figure S23 shows plots from the isotherm fitting.

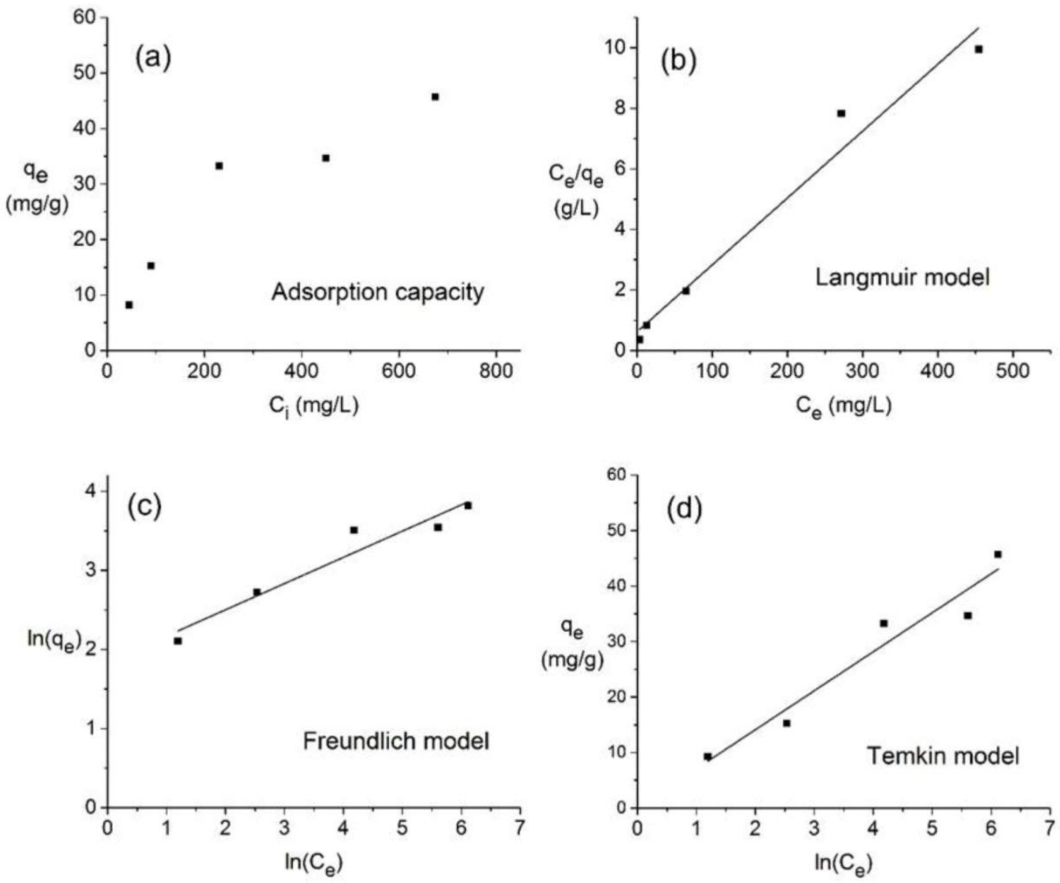

Figure S23: Equilibrium isotherm models for adsorption of MB from acetonitrile at 333 K. (a) Adsorption capacity from different initial concentrations, (b) Langmuir model, (c) Freundlich model, and (d) Temkin model.

Kubelka-Munk representation of DRS data

Figure S24 shows linear fits to diffuse reflectance spectroscopy data.

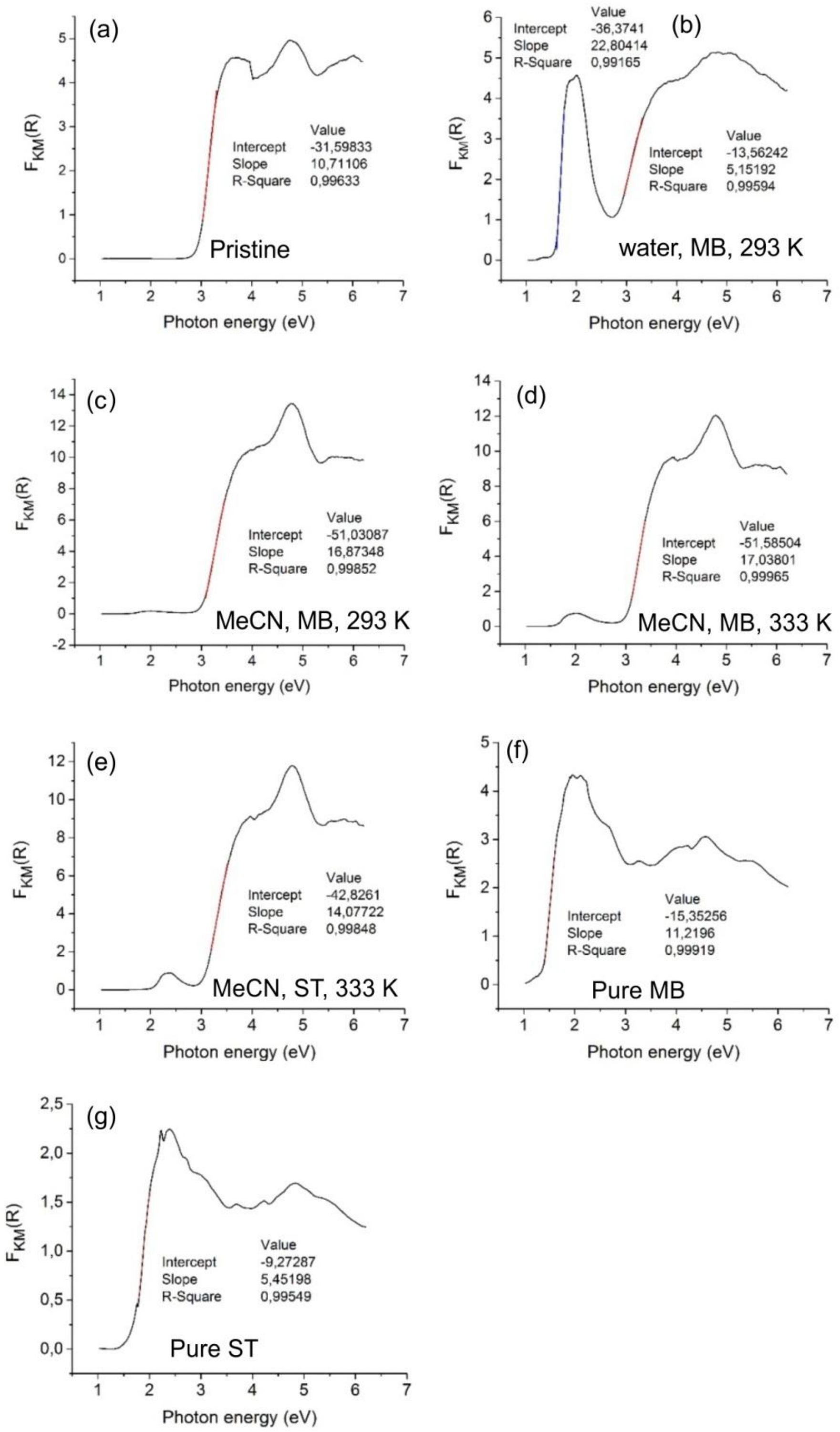

Figure S24: Kubelka-Munk representation of diffuse reflectance data of (a) Pristine $Sn_3S_7(trenH)_2$, samples stained from (b) 230 mg L^{-1} MB/H_2O, 293 K, (c) 230 mg L^{-1} MB/MeCN 293 K, (d) 230 mg L^{-1} MB/MeCN 333 K, (e) 230 mg L^{-1} ST/MeCN 333 K, (f) pure MB, (g) pure ST.

Effect on light absorption of an SnO_2 impurity

The effect of an SnO_2 impurity on the light absorption properties of $Sn_3S_7(trenH)_2$ was studied by mixing a known amount of SnO_2 into a sample of as-synthesized $Sn_3S_7(trenH)_2$ and by subsequently measuring diffuse reflectance spectroscopy data on these samples. Experiments with 10 and 20 wt% added SnO_2 were compared to pure SnO_2 and the as-synthesized $Sn_3S_7(trenH)_2$. The small change in the reflectance data reveals that an impurity of SnO_2 only has a small effect on the light absorption data.

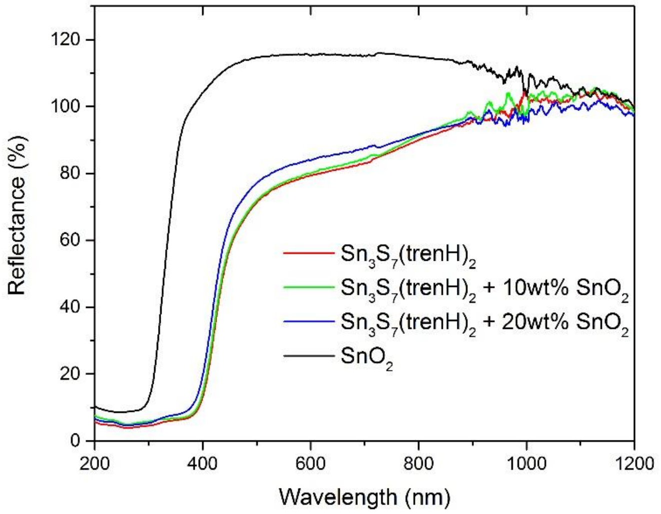

Figure S25: Diffuse reflectance spectra of $Sn_3S_7(trenH)_2$, SnO_2, and $Sn_3S_7(trenH)_2/SnO_2$ mixtures.

1 Filsø M., Chaaban, I., Al Shehabi, A., Skibsted, J. & Lock, N. The template changes everything: Structures and optical properties of two-dimensional thiostannates (in review, 2016)

2 Pienack, N., Schinkel, D., Puls, A., Ordolff, M.-E. & Luehmann, H. New Thiostannates Synthesized Under Solvothermal Conditions: Crystal Structures of (trenH)2Sn3S7 and {[Mn(tren)]2Sn2S6}. *Zeitschrift für Naturforschung. B, A journal of chemical sciences* **67**, 1098-1106, doi:10.5560/znb.2012-0126 (2012).
